# Cytological and Ploidy Analyses Revealing Fertility Defects of Cultivar *Cyperus esculentus* L. in China

**DOI:** 10.3390/plants14223431

**Published:** 2025-11-10

**Authors:** Meng Wang, Ke Hu, Yan Wang, Yilin Tian, Xiaoying Du, Jiaying Zhu, Huiwei Wang, Chunxin Li, Pingli Lu

**Affiliations:** 1 State Key Laboratory of Crop Stress Adaptation and Improvement, School of Life Sciences, Henan University, Kaifeng 475004, China; wangm@henu.edu.cn (M.W.); huke20191213@163.com (K.H.);; 2 Institute of Industrial Crops, Henan Academy of Agricultural Sciences, Zhengzhou 450002, China

**Keywords:** *Cyperus esculentus* L., anther, fertility, pseudomonad, ploidy

## Abstract

*Cyperus esculentus* L., commonly known as Yellow Nutsedge or Tiger Nut, belongs to the Sedge family (Cyperaceae), which is widely distributed in tropical and temperate regions around the world. The species is particularly noted for its underground tuber, which is rich in starch and oil, and can be used for both food and industrial purposes. However, cultivated *C. esculentus* rarely flowers in the field, and when it does, flowering is often accompanied by sterility, significantly hindering the development of superior varieties through sexual hybridization. Our investigation into the causes of pollen abortion in *C. esculentus* yielded several key findings. Initially, we observed pollen abortion within the anthers using Alexander’s red staining and starch-iodide staining techniques. Further examination through semi-thin sections of the anthers revealed that, although the pollen appeared normal during the pachytene stage, the pollen failed to stain blue in later developmental stages, indicating abnormal development. This was followed by vacuolation, collapse, and degradation of the pollen, ultimately resulting in abortion. Additionally, we monitored chromosome behavior throughout meiosis and observed premature chromosome separation during metaphase II, leading to disarray in the subsequent pseudomonad phase. Chromosomal karyotype analysis and ploidy predictions suggested that the triploid nature of *C. esculentus* contributes to meiotic disorders, which may explain the phenomenon of pollen abortion. These findings provide valuable insights into the reproductive challenges faced by this species and may inform future breeding efforts aimed at overcoming its sterility.

## 1. Introduction

*Cyperus esculentus* L., commonly known as the tiger nut, is an herbaceous perennial C_4_ plant belonging to the Cyperaceae (sedge) family within the Poales order [1,2]. Renowned for its high yield, substantial oil yield, and versatile potential for utilization, it is cultivated widely in the world [3,4,5]. Historical records suggest that tiger nuts were first domesticated by ancient Egyptians, who utilized them for both culinary and medicinal purposes [6,7]. In modern times, *C. esculentus* has undergone global dissemination [8,9]. During the mid-19th century, it was introduced to the southern United States and South American nations, including Chile and Brazil, where it was primarily utilized as forage [10,11]. In the early 1930s, the Soviet Union introduced *C. esculentus* and conducted cultivation trials, which first demonstrated its potential as a high-yield oil crop in northern temperate zones [12]. It is noteworthy that five varieties of *C. esculentus* have been identified, comprising four wild varieties (*C. esculentus* var. *esculentus*, var. *heermannii* (Buckley) Britton, var. *leptostachyus* Boeckeler, and var. *macrostachyus* Boeckeler) and one cultivated variety (var. *sativus* Boeckeler) [2]. The variety widely cultivated in China was thought to be introduced from the Soviet Union and corresponds to the cultivated variety (var. sativus Boeckeler). However, this variety might have undergone unknown genetic changes during cultivation and breeding. Currently, studies on *C. esculentus* in China are primarily focused on its developmental traits, growth habits, nutritional composition, cultivation practices, and the mechanisms of oil accumulation [13,14,15].

As we know, so far, *C. esculentus* is unique among sedge species due to its ability to store large quantities of oil in its underground tubers. It was reported that the tubers contain about 22–45% oil, 23–48% starch, 3–8% protein, and 8–15% fiber [7]. Notably, its oil is particularly rich in oleic acid (up to 75%), comparable to olive oil, which contains about 73%, thereby positioning tiger nut as a promising source of high-quality edible oil [16,17]. *C. esculentus* has been considered a valuable oil crop with significant agricultural potential.

However, the species faces major challenges in terms of breeding. *C. esculentus* propagates asexually Via underground tubers, resulting in limited genetic diversity. In clonally propagated species, inbreeding depression tends to be more severe than in sexually propagated plants. This is primarily due to the accumulation of deleterious or harmful mutations, which cannot be efficiently purged in the absence of genetic recombination during meiosis [18]. As the cultivar species usually fail to complete the functional sexual reproduction, it greatly hinders the conventional breeding program. The exclusive reliance on asexual propagation Via tubers leads to severe genetic uniformity. Over time, this clonal propagation strategy could result in progressive varietal degeneration.

Notably, purple nutsedge (*Cyperus rotundus* L.), as a close relative of yellow nutsedge (*Cyperus esculentus* L.), shares similar growth and developmental characteristics [13,14]. Phylogenetic analysis has confirmed the close evolutionary relationship between *C. esculentus* and *C. rotundus* [19]. Both belong to the genus Cyperus within the Cyperaceae family, possessing similar morphological features, such as producing tubers [20]. However, unlike the low fertility and poor seed-setting rate of cultivated *C. esculentus*, *C. rotundus* can flower normally and produce viable seeds under natural conditions [21]. Considering these similarities and differences, we believe that *C. rotundus* can be used as a useful control species for investigating the fertility defect of *C. esculentus*.

In flowering plants, seed formation is a complicated process involving the development of male and female gametophytes, followed by double fertilization [22]. Male gametophyte development involves the differentiation of meiocytes (pollen mother cells), which undergo meiosis to produce haploid microspores (mononuclear pollen grains). These microspores then undergo mitosis to form tricellular pollen grains [23]. Pollen development occurs in the anther and involves intricate coordination between various anther tissues [24]. Typically, angiosperm anthers are tetrasporangiate and comprise four distinct wall layers: epidermis, endothecium, middle layer, and apetum [25]. Key stages of anther development include archesporial cell differentiation, meiosis of pollen mother cells, regulation of tapetum function, and anther wall dehiscence. Disruption in any of these processes can lead to male sterility [21].

Morphological staging systems for anther development have been well characterized in certain species, such as *Arabidopsis thaliana* [26], *Oryza sativa* [27], *Triticum aestivum* [25], and *Brachypodium distachyon* [24]. The onset of anther development is marked by the formation of archesporial cells, which asymmetrically divide into two cell lineages: primary parietal cells and primary sporogenous cells [24]. Subsequent stages range from the emergence of the tapetum (Stage 5, pre-callose phase) to the formation of mature pollen grains and tapetum degeneration (Stage 12, trinucleate pollen phase) [25]. During microsporogenesis, the vacuolated microspore undergoes asymmetric mitosis, producing a smaller generative cell and a larger vegetative cell with a single nucleus. In stage 12, the generative cell divides again to yield two sperm cells, resulting in a mature trinucleate pollen grain with two compact sperm nuclei and a prominent vegetative nucleus—an essential structure for double fertilization in angiosperms [27].

Unlike the symmetric meiosis typical of most angiosperms, sedges undergo a distinctive process called asymmetrical meiosis during microsporogenesis. This process produces a coenocytic structure with four nuclei, three of which degenerate, leaving a single functional nucleus. Upon cell wall formation, the degenerating microspores encase the three non-functional nuclei, while the viable nucleus forms a pseudomonad structure [28,29]. Although degenerating microspores may initiate mitosis, they ultimately fail, whereas the functional microspore successfully progresses to form a tricellular pollen grain [29,30]. Despite advances, our understanding of anther development and meiosis in the Cyperaceae family remains incomplete.

In China, cultivated *C. esculentus* is more and more widely used in agriculture, especially for improving desert fields. However, to date, the severe fertility defect under normal growth conditions and the underlying cytological basis still remain poorly understood. Meanwhile, studies on the reproductive characteristics of its close relative, purple nutsedge (*C. rotundus*), are also lacking. In this study, we aim to elucidate the cytological mechanism affecting fertility defects in *C. esculentus* by comparatively analyzing the cytological characteristics and fertility of cultivated *C. esculentus* and *C. rotundus*. We employed Alexander staining and iodine-potassium iodide staining to assess pollen viability and starch accumulation in both species; utilized semi-thin sections to systematically investigate cytological changes during the anther development; applied meiotic chromosome spread technique to analyze chromosomal behavior at various meiosis stages; and conducted karyotype analysis and ploidy identification to determine the chromosome number and genomic characteristics of *C. esculentus*. These findings provide direct cytological evidence for clarifying the cellular basis of fertility defects in the cultivated *C. esculentus*, thereby laying a solid scientific foundation for its subsequent genetic improvement and efficient breeding strategies.

## 2. Results

### 2.1. Seed Set and Fertility of C. esculentus

**Figure 1:**
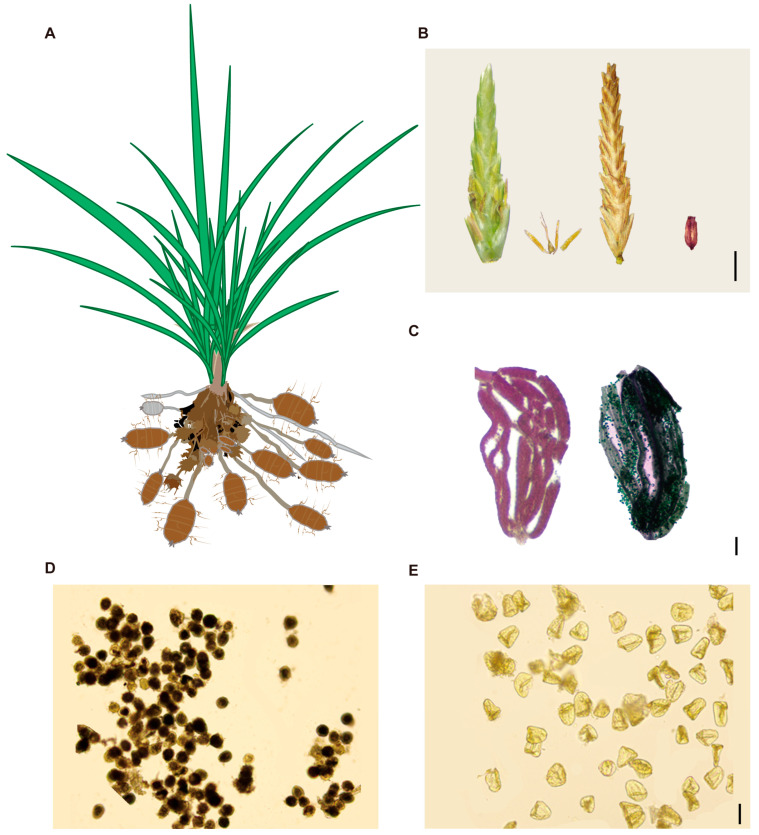
(**A**) Schematic diagram of *Cyperus esculentus* plant. (**B**) Dissection diagram of a single Cyperus esculentus flower, from left to right, immature flower, anthers and ovary within the anther, and stigma, mature flower, empty seed, scale bar is 2 cm. (**C**) Left is Alexander’s red staining of Cyperus rotundus anthers, right is Alexander’s red staining of anthers, scale bar is 0.5 mm. (**D**) Iodine-potassium starch staining of pollen from *Cyperus rotundus*, scale bar is 0.1 mm. (**E**) Iodine-potassium starch staining of pollen from *Cyperus esculentus*, scale bar is 0.1 mm.

The aerial parts of *Cyperus esculentus* resemble the grasses in Poaceae: its leaves are long and narrow, while the tips of its subterranean stolons swell into bean-like tubers that serve as the main storage organs (Figure 1A). Each floret bears three stamens and a pistil with three stigmas. The developed mature seed is trigonous (Figure 1B). To assess male reproductive function, Alexander red staining was first performed to examine pollen activity. In *C. esculentus*, pollen inside anthers was stained green (Figure 1C, left), indicating non-viability, whereas that of *C. rotundus* was stained red (Figure 1C, right), signifying viable, active pollen. Further assessment using starch-potassium iodide staining revealed that *C. esculentus* pollen grains were largely hollow and remained unstained, indicating an absence of starch accumulation and viability (Figure 1D). In contrast, *C. rotundus* pollen grains stained darkly, showing starch accumulation and viability (Figure 1E).

Seed-set analysis of 13 occasionally flowering *C. esculentus* individuals in the field revealed that 99.9% of the seedpods observed were empty, lacking viable embryos (Figure 1B). On rare occasions, a few seeds appeared present but were found to be collapsed and immature. These results suggest that *C. esculentus* experiences pollen abortion, leading to reproductive failure and a near-complete absence of viable seed formation. This reproductive limitation likely underlies the species’ reliance on asexual propagation through tubers and represents a significant barrier to conventional breeding efforts.

### 2.2. Semi-Thin Section Analysis of Anther Development in C. esculentus and C. rotundus

To explore anther development process and determine the developmental stage at which abnormalities arise in *C. esculentus* anthers, semi-thin cross-sections were prepared and compared to those of *C. rotundus*. Anther development in flowering plants involves a series of cellular differentiation and tissue organization. In this study, eight distinct developmental stages were identified through bright-field microscopy of stained sections. Anther length associated with different development stages was also included in the reproductive development of *C. esculentus* (Table 1).

**Figure 2:**
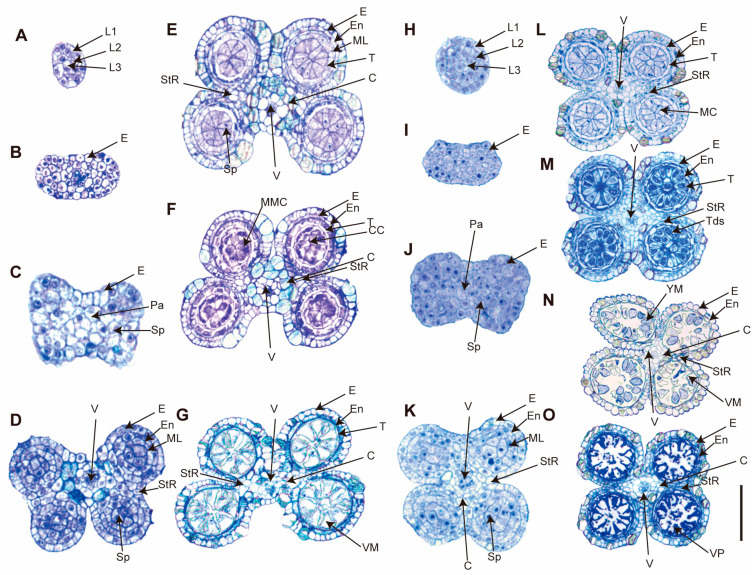
Anther development of *C. esculentus* and *C. rotundus* in each stage of semi-thin section analysis. (**A**–**G**) show A cross-section of various stages of anther development in *C. rotundus*. (**H**–**O**) are a cross-section of various stages of anther development in *C. esculentus*. After the growth of *C. esculentus* and *C. rotundus* was bolted for a month, the inflorescence was taken and fixed with FAA solution to make resin. Anther development was observed by semi-thin section with a scale of 50 μm. L1, 1st Cell layer; L2, 2nd Cell Layer; L3, 3rd Cell layer; Pa, Parietal Tissue; Sp, Sporogenous Tissue; E, Epidermis; En, Endothecium; ML, Middle Layer; T, Tapetum; StR, Stomium Region; MMC, Microspore Mother Cells; Tds, Tetrads; YM, Young Microspores; VM, Vacuolate Microspores; VP, Vacuolate Pollen; MC, Meiotic Cells; V, Vascular Region; C, Connective Tissue; CC, Central Callose.

During the early stages (stages A–D, Figure 2), the anthers of *C. esculentus* developed normally, with well-defined layers of epidermis, endothecium, middle layer, and tapetum, giving the anther its characteristic structure with four distinct locules. By stage E, referred to as the central callose stage, microspore mother cells had differentiated from the sporogenous ones. This is followed by meiosis at stage F, marking a critical phase in the anther development. At stage G, characterized as the vacuolate microspore stage, the middle layer and tapetum begin to degrade sequentially. During this stage, microspores mature and accumulate sugars and other nutrients, which are released into the locular cavity through Ubisch bodies. At stage M, microspores develop to a bi-nucleate state. By stage N, the tapetal layer is almost entirely degraded, and the pollen grains become filled with starch, marking the final stages of pollen development.

In *C. rotundus*, pollen development progressed normally throughout all stages (Figure 2A–G). However, significant abnormalities were observed in *C. esculentus* during the later stages of anther development. While the initial stages of anther development in *C. esculentus* appeared normal, abnormalities began to emerge during meiotic and post-meiotic stages. The pollen was stained in blue, indicative of defective development with vacuolation (Figure 2N). Subsequently, the pollen collapsed, degraded, and shriveled (Figure 2O), ultimately leading to complete abortion. Due to the transient and indistinct nature of the post-meiosis abortion stage in *C. esculentus* anthers, repeated attempts to sample and analyze this phase have been unsuccessful in yielding complete datasets; however, these shortcomings do not compromise the conclusions drawn in this study. These observations suggested that developmental defects arise during or shortly after meiosis in *C. esculentus*.

### 2.3. Meiotic Chromosomal Behavior in C. esculentus and C. rotundus

**Figure 3:**
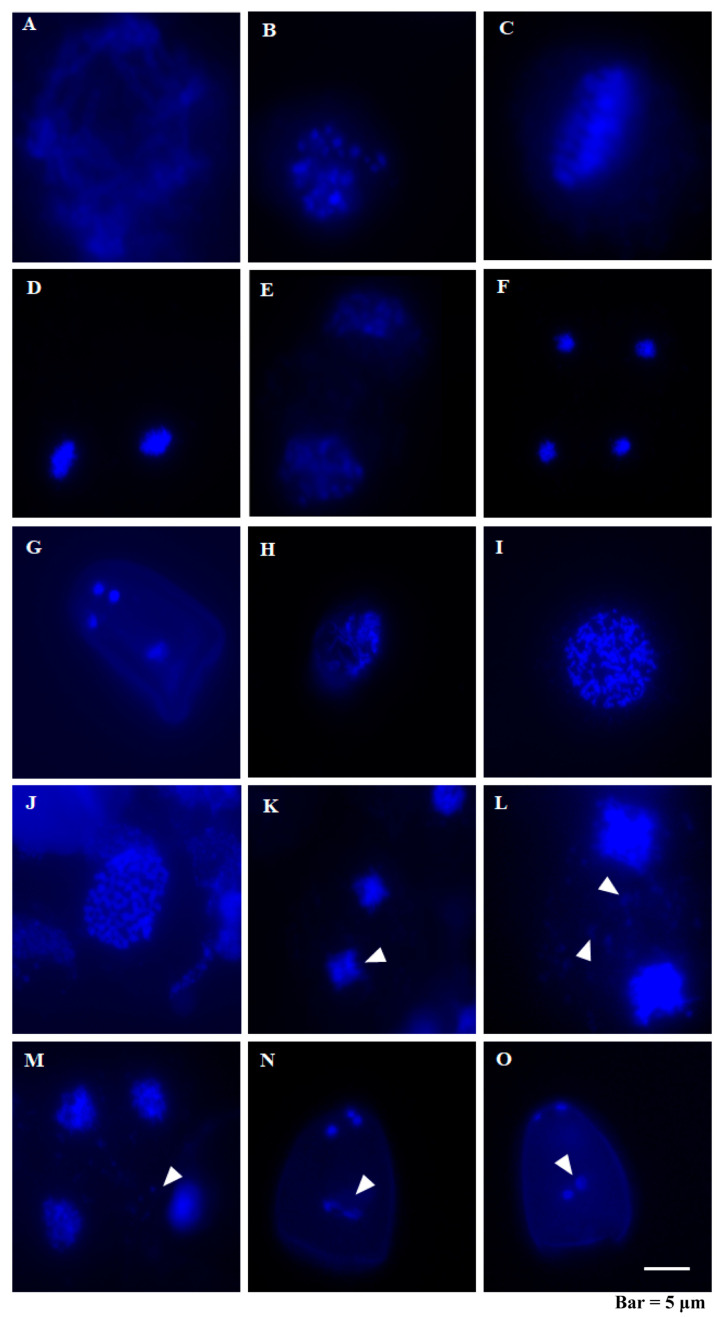
Analysis of meiosis chromosome behavior in different periods of *C. esculentus* and *C. rotundus*. (**A**–**G**) are the images of chromosome behavior during the various stages of meiosis in Cyperus rotundus, and (**H**–**O**) are also the images of chromosome behavior during the various stages of meiosis in *C. rotundus*. The anther of the meiotic period was taken for fixation, and the chromosome spread. Scale bar is 50 μm. (**A**) Pachytene; (**B**) Diakinesis; (**C**) Metaphase I; (**D**) Telophase I; (**E**) Prophase II; (**F**) Anaphase II; (**G**) pseudomonad; (**H**) Diplotene; (**I**) Diakinesis; (**J**) Metaphase I; (**K**) Telophase I; (**L**) Prophase II; (**M**) Telophase II; (**N**) pseudomonad; (**O**) pseudomonad.

As meiosis plays a direct role in creating haploid gametes for double fertilization in flowering plants, we investigated meiotic chromosome behaviors during meiosis in *C. esculentus* to see if meiosis is normal or not. Given that *C. rotundus* can normally produce seeds, it is supposed to perform successful meiosis. During pachytene, synapsis is fully established, and chiasmata, indicative of recombination, begin to form, with recombination continuing to progress (Figure 3A). In diplotene, the synaptonemal complex disassembles, and homologous chromosomes remain connected by crossovers (Figure 3H). At diakinesis, the bivalents condense further, marking the culmination of prophase I, and the nuclear envelope breaks down (Figure 3B,I). Metaphase I is defined by the alignment of homologous chromosomes along the equatorial plate with their centromeres symmetrically positioned toward opposite poles (Figure 3C,J). During telophase I, the two sets of chromosomes congregate at the two poles of the cell, forming haploid nuclei. The nuclear envelopes reform, and the nucleoli reappear (Figure 3D,K). In Prophase II, chromosomes align at the equator; meanwhile, the nuclear envelope breaks down once again (Figure 3E,L). Anaphase II involves the separation of chromosome centromeres, with sister chromatids migrating to opposite poles (Figure 3F). At telophase II, the cell divides to form four new nuclei, and the nuclear envelope reforms (Figure 3M). In *Arabidopsis thaliana*, meiosis results in four equal daughter cells, which undergo two rounds of mitotic divisions to produce mature trinucleate pollen [31]. However, it was reported that plants in the Sedge family exhibit a different pattern, in which meiosis ends with the degeneration of three nuclei, leaving a single functional nucleus that forms pseudopollen [1]. In this study, we observed significant abnormalities in *C. esculentus* meiosis. We observed abnormal premature chromosomal separation during Telophase I (*n* = 28, Percentage of abnormal 82.14%) (Figure 3K). The chromosomes of *C. esculentus* are excessively small. During meiosis, a substantial amount of genetic material is observed in the central region of the cell at prophase II and telophase II, displaying irregular and scattered distribution, which is speculated to be micronuclei and unpaired chromosomes. This phenomenon is not observed in the meiosis process of *C. rotundus* (Figure 3L,M). During the process of pseudopollen formation, abnormal phenomena were observed, including the development of pollen grains with more than four nuclei (Figure 3N) and the presence of two central nuclei (Figure 3O). These disruptions in meiosis and pseudopollen separation likely contribute to the formation of pollen abortion observed in *C. esculentus*.

### 2.4. Chromosome Number and Ploidy Analysis of C. esculentus

**Figure 4:**
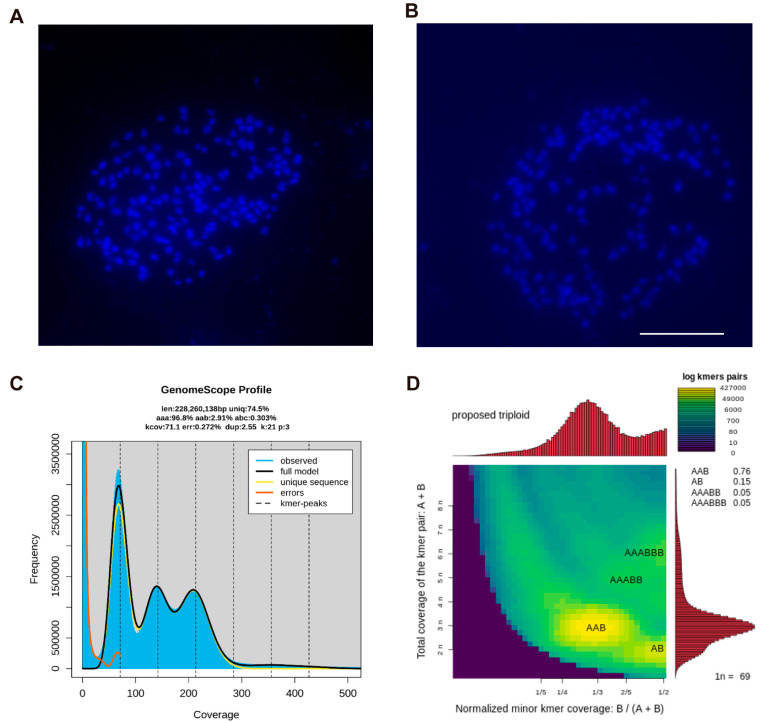
Chromosome number detection and ploidy analysis of *C. esculentus*. Blue signals on the black background indicate chromosomes. The root tip of a week of hydroponics was selected as the experimental material, the length of which was 1 cm. (**A**,**B**) Mitotic exhibits of the root tip of the bean, 20 times of data statistics, two representative images were selected for display; bar = 50 μm. (**C**) CNA0051961 Genome data Selected as raw data for analysis, genomic data survey using jellyfish. Generating genome ploidy map. (**D**) Ploidy stain map of the genome of *C. esculentus*.

To determine the chromosome number of *C. esculentus*, a specialized method was developed for chromosome analysis. Karyotype analysis was performed on the root tips of *C. esculentus*, a region exhibiting vigorous mitotic activity, using a modified hypotonic treatment and cell wall removal method. Karyotype analysis revealed that the somatic chromosome number of *C. esculentus* was 2*n* = 162, with uniformly minute chromosomes measuring 0.6 to 1 μm in length. Due to their exceedingly small size and high morphological uniformity, key diagnostic features such as centromere positions are hardly reliably discernible. Chromosomes could not be accurately paired, ordered, or classified according to conventional cytogenetic standards, preventing the construction of a definitive karyotype (Figure 4A,B). It was suggested that the haploid chromosome numbers among various Sedge species exhibit considerable diversity, with reported counts of 8, 18, 32, 36, 40, 48, 52, 54, and 56 [32]. Researchers have discovered that *Carex cilicica* has a chromosome number of 54 [33], equivalent to one-third of the chromosome number in the cultivar *C. esculentus*. This result implied that the currently cultivated variety in China is likely a triploid species.

To confirm the above conclusion, further genomic analysis was performed. First, the raw genome sequencing data for *C. esculentus* were obtained from the national genomic library (https://db.cngb.org/search/assembly/CNA0051961/ accessed on 12 December 2022) [34]. Secondly, using the Jellyfish **3.0** software, a K-mer analysis was used to generate a frequency distribution table, which was processed through GenomeScope 2.0 for genome characterization [35]. Finally, a heatmap was generated using the Smudgeplot v0.2.5 software. Our analysis confirmed that the *C. esculentus* we used in this study is a triploid species (*p* = 3, Figure 4C). Moreover, the genomic analysis showed that the AAB genotype had the highest distribution rate, strongly supporting that *C. esculentus* possesses a triploid genome with an AAB configuration (Figure 4D). These findings provide a comprehensive understanding of the chromosomal behavior, ploidy characteristics, and genome composition of *C. esculentus*, shedding new light on the genetic mechanisms underlying its reproductive challenges and evolutionary adaptations.

## 3. Discussion

The Cyperaceae family, commonly known as sedges, represents the third-largest monocot family, comprising approximately 5500 species [31]. As we know so far, among these, *Cyperus esculentus* is unique in its ability to store significant quantities of oil within its underground tubers. In China, however, *C. esculentus* is predominantly propagated via its tubers, costing a great deal of valuable materials. But its grain size varies significantly, varieties are often mixed, and its commercial value remains low. Its cultivation via seeds is still facing several challenges, including a lack of commercially viable, high-quality varieties and inconsistent yield and quality among existing strains. To solve these limitations, it is required to deeply understand its reproductive biology, particularly the molecular mechanisms affecting seed development, which is a critical barrier to crossbreeding and genetic improvement.

Seed abortion is a widespread phenomenon observed in numerous plant species, including major crops such as rye, maize, and soybeans. When parents with different ploidy levels or distant genetic backgrounds are crossed, viable seeds often fail to develop due to multiple reproductive progress defects, such as abnormal meiotic recombination and embryo abortion, complicating traditional crossbreeding strategies [36,37,38]. The underlined mechanisms of seed abortion typically fall into four categories: (1) male sterility, including male game development defect, anther degeneration, pollen abortion, and structural/functional abnormalities in the tapetum [39,40,41]; (2) female organ infertility, characterized by abnormal morphology, degeneration of megaspore mother cells, and dysfunction of vegetative organ [42,43,44,45]; (3) pollination and fertilization defects, often due to self- and cross-incompatibility [46,47,48]; and embryo abortion, with varying timing and anatomical features across species [49,50]. Male sterility is particularly well-studied and is often utilized in hybrid seed production [51,52]. Pollen abortion is generally caused by meiotic disorders in pollen mother cells, chromosomal abnormalities, or tapetum development defects [39,40]. Male sterile plants can usually be used to generate hybrid seeds to meet the increasing global food demand [53]. In this study, we primarily investigated the development of the male gametophyte to elucidate the potential causes of reproductive abortion in *C. esculentus*. Certainly, the development of the embryo sac, pollination, double fertilization, and others also play indispensable roles in fertility. To establish a more comprehensive understanding of *C. esculentus* reproductive biology, it is absolutely necessary to investigate the above processes in future studies.

We noticed that the cellular mechanism controlling seed abortion in *C. esculentus* displayed distinct characteristics from those of other model species, like Arabidopsis and rice. One notable feature is the formation of pseudopollen, which has been attributed to asymmetrical meiosis. During this process, one central functional microspore further develops, whereas the other three peripheral microspheres degenerate [30,54]. Despite these distinctive traits, we also found that *C. esculentus* had additional abnormalities during late pollen development stages, marked by vacuolization, collapse, and degradation, ultimately resulting in pollen abortion. Disruptions are also evident during pseudopollen formation, with observations of more than four nuclei and disorganized separation (Figure 3N,O), deviating from the typical pattern. Further analysis of meiosis in *C. esculentus* revealed that the chromosomes are delayed during metaphase of the second meiotic division. However, no disordered chromosomes were observed during anaphase I of meiosis, which appears to differ from the meiotic behavior typically seen in triploids. We speculate that this may be related to the holocentric chromosome characteristic of Cyperaceae, where each chromosome possesses multiple diffuse centromeres [55,56]. Research has demonstrated that the holocentric chromosomes in Rhynchospora undergo significant structural reorganization during the transition from somatic to meiotic cells, with their centromeric signals no longer exhibiting a longitudinal distribution [57,58]. For a triploid with such multicentric chromosomes, the mechanisms governing chromosome pairing and segregation remain unclear.

Based on combined karyotype analysis and genome ploidy prediction, we hypothesize that the cultivated *C. esculentus* is triploid. It was reported that *C. rotundus* is a diploid (aa) with a haploid chromosome base number of x = 55 and a somatic chromosome number of 2n = 110 [59]. These findings suggest that *C. rotundus* has a relatively stable genetic background and is capable of producing fertile pollen. Triploidy is well-documented to disrupt normal meiotic processes, often resulting in obvious irregularities in chromosome pairing and segregation, such as the formation of multivalents or univalents, and chromosome bridges during meiosis [60,61,62]. Therefore, the doubling technique offers a promising approach to alleviating the adverse effects of triploidy by restoring the normal meiotic process and increasing the success of reproduction. Chromosome doubling also enables the fixation and propagation of desirable genetic traits from sterile triploid plants, paving the way for new cultivar development. Furthermore, this technique can enrich the genetic diversity of *C. esculentus* and significantly accelerate the breeding process. Many of the world’s staple food crops and economically important species are polyploids [63]: hexaploid sugarcane (*Saccharum officinarum*), the highest-yielding cultivated plant, contributes 80% of global sucrose production [64]; hexaploid common wheat (*Triticum aestivum* L.) supplies about 30% of human dietary calories and 20% of protein [65]; tetraploid cultivated potato (*Solanum tuberosum* L.) serves as a staple food for approximately 1.3 billion people worldwide [66]. An important application of polyploidy in modern breeding is the doubled haploid (DH) technology, which involves generating haploid plants followed by chromosome doubling to rapidly achieve homozygous inbred lines, significantly shortening the breeding cycle [67]. Studies have shown that artificially induced polyploidy is an important tool in plant breeding, capable of enhancing crop yield and biomass [68]. Due to their larger cell and organ size, polyploid plants generally demonstrate higher biomass [68]. Furthermore, polyploidization can strengthen plant resistance to biotic and abiotic stresses such as diseases, pests, drought, and salinity [68].

In conclusion, the unique reproductive features of *C. esculentus*, including pseudopollen formation and triploid-related meiotic abnormalities, present significant challenges to its fertility. Pollen abortion in *C. esculentus* is primarily driven by early chromosome separation during the meiotic metaphase II and disorganized separation at the pseudopollen stage. Chromosome karyotype and genomic analyses further showed that *C. esculentus* used is triploid, with its meiotic disorders directly linked to pollen abortion. These findings provide critical insights into the species’ reproductive biology and lay the foundation for future genetic improvement efforts.

## 4. Materials and Methods

### 4.1. Plant Material

The *C. esculentus* used in this study was the cultivated variety named “YuYousha 7” (variety right number: 2024005), bred by the Henan Academy of Agricultural Sciences and propagated asexually Via tubers. The field planting population size was approximately 600 individuals, located in Kaifeng, Henan Province, China (34°48′36″ N, 114°18′36″ E). The growth period spanned from May to October each year, with sampling conducted in July. The annual sampling was repeated for three consecutive years. The *C. rotundus* material was of a wild type (*C. rotundus* var. *quimoyensis*), also propagated asexually through tubers, with a wild population of approximately 1000 individuals. The sampling location, time, and methods were consistent with those used for *C. esculentus*. Furthermore, to facilitate controlled environmental conditions and repeated sampling, both *C. esculentus* and *C. rotundus* were cultivated in a greenhouse, with population sizes of 30–50 individuals each. The greenhouse conditions were set at a constant temperature of 25 °C and a photoperiod of 16 h of light and 8 h of darkness. A total of 37 flowering plants of *C. esculentus* were obtained, with 36 collected from the field and 1 from the greenhouse. Approximately 30 florets per plant were sampled for subsequent studies. For *C. rotundus*, 83 flowering plants were acquired, comprising 80 from the field and 3 from the greenhouse.

### 4.2. Alexander Staining of Anthers

For the Alexander red staining assay, samples were taken from 6 cultivated individuals of *C. esculentus* from one population, comprising 60 florets (approximately 240 anthers), and from 12 wild individuals of *C. rotundus* within a single population, comprising 96 florets (approximately 290 anthers). Flowers were fixed in a centrifuge tube containing Carnoy’s fixative for a minimum of 4 h. After fixation, the flowers were carefully removed using tweezers and transferred to a 1.5 mL centrifuge tube containing Alexander’s stain. The samples were incubated in a 60 °C oven for 3 to 5 h. Subsequently, the flowers were then peeled under a dissecting microscope. To clean the anthers, 10 μL of sterile water was added, and a cover slip was gently placed on top to flatten the anthers. Another 10 μL of Alexander’s stain was added to the center of the slide [69,70]. The fully opened flowers were gently shaken to release pollen onto the stain. A cover slip was applied, and after a brief incubation, the samples were observed and photographed under a dissection microscope.

### 4.3. Starch-Potassium Iodide Staining

For this experiment, samples from six cultivated *C. esculentus* individuals from a single population, comprising 30 florets (approximately 150 anthers), and from ten wild *C. rotundus* individuals within a single population, comprising 60 florets (approximately 200 anthers), were used. Mature anthers from *C. esculentus* and *C. rotundus* were collected and crushed with tweezers to release pollen grains. The pollen was treated with Lugol’s solution, covered with a cover slip, and observed under a microscope [71].

### 4.4. Meiotic Chromosome Spreading Experiment in C. esculentus and C. rotundus

For this experiment, samples from 12 cultivated *C. esculentus* individuals of a single population, comprising 90 florets (approximately 320 anthers), and from 12 wild *C. rotundus* individuals of a single population, comprising 96 florets (approximately 400 anthers), were used. Whole inflorescences were fixed in Carnoy’s fixative, washed with water, and placed on a slide. anthers were carefully dissected under a microscope using a dissection needle. Remaining inflorescence tissues were transferred to a centrifuge tube containing cell lysis buffer and incubated at 37 °C for 1 h. Anthers measuring 0.3–0.4 mm were isolated under a microscope, and dehydration was complete. A 5 μL drop of DAPI was added to the center of the sample, and a cover slip was applied. After 5 min, the sample was examined under a fluorescence microscope [72].

### 4.5. Semi-Thin Section Experiment in C. esculentus and C. rotundus

For this experiment, samples comprising 120 florets (approximately 480 anthers) from 12 cultivated *C. esculentus* individuals of one population, and 140 florets (approximately 560 anthers) from 12 wild *C. rotundus* individuals of a single population, were used. Flower buds were fixed in FAA solution and subjected to vacuum treatment three times, each for 1 h. The samples were dehydrated stepwise with increasing ethanol concentrations (50%, 60%, 70%, 80%, 90%, and 95%) for 30 min per concentration. Two rounds of dehydration with absolute ethanol were performed, each for 30 min. Ethanol was then replaced with acetone at increasing concentrations (the concentration ratios of ethanol to formaldehyde are 2:1, 1:1, and 1:2), with 30 min intervals for each ratio. Pure acetone was applied twice for 30 min each. Acetone was subsequently replaced with Spurr resin at increasing concentrations (the concentration ratios of acetone to resin are 2:1 and 1:1, for 2 h each), followed by immersion in pure resin twice for 12 h each [73].

The buds were embedded in Spurr resin on embedding plates, ensuring no bubbles. Additional resin was added, and the samples were polymerized in a 70 °C incubator for 12 h. Resin blocks were sectioned into 2 μm slices using a glass blade and transferred to slides pre-wetted with water. The slides were dried on a 70 °C hotplate and stained with 0.1% toluidine blue for 1 min, followed by rinsing with distilled water and drying. Samples were observed, and photographs were collected under an optical microscope [73].

### 4.6. Karyotype and Ploidy Analysis of C. esculentus

Approximately 120 tubers from 30 plants of a single *C. esculentus* population were placed in a 40 °C incubator for hydroponic germination in the dark until the root length reached approximately 2 cm. Root tips (~5 mm) were fixed in freshly prepared Carnoy’s fixative. After fixation, the root tips were rinsed and treated with 1 mL 75 μM KCl solution for 20 min for hypotonic treatment. The low-permeability root tips were sectioned (~2 mm of meristem tissue) and placed in a centrifuge tube containing enzymatic hydrolysate. Enzymatic digestion was performed at 37 °C for 1 h. After digestion, the root tips were treated with distilled water for 10 min, and the dissociated root tips were pipetted to obtain a cell suspension [74].

Sterile water was added to the suspension, and the samples were centrifuged at 6000 rpm for 1 min twice. A 50 μL aliquot of cell suspension was dropped onto a slide and dried on a 42 °C graphite heating plate. Dried slides were stained with 3 μL DAPI fluorescent dye in the dark. Five cells with clear, non-overlapping chromosome morphology were selected for karyotype analysis under a 100× oil immersion lens [74].

Ploidy analysis was performed using whole-genome sequencing data of *C. esculentus* obtained from the National Genomics Data Center (https://db.cngb.org/search/assembly/CNA0051961/, accessed on 12 December 2022) [34]. K-mer frequency distribution was generated from the raw sequencing data using Jellyfish, followed by genome characterization with GenomeScope. Finally, a Smudgeplot analysis was conducted to further resolve the ploidy structure [35].

## 5. Conclusions

Using Alexander staining, semi-thin sectioning, and live imaging of meiosis, we demonstrated that microspore development in tiger nut proceeds normally until the pachytene stage but is subsequently arrested through vacuolization and collapse. This abortion is triggered by premature chromosome segregation at metaphase II, which disrupts the formation of functional pollen. Karyotype and ploidy analyses consistently identified the plant as triploid, which is likely the primary cause of meiotic failure, thus providing a theoretical foundation for overcoming this reproductive barrier.

## Figures and Tables

**Table 1:** Anther length range for anthers from stage H to stage 0.

Anther Stage	Stage Name	Range (mm)
H	Stamen primordia	0.1–0.15
I	Archesporial	0.2–0.4
J	Sporogenous tissue	0.3–0.45
K	Lobe formation	0.4–0.6
L	Pre-callose	0.5–0.75
M	Meiotic	0.6–0.9
N	Pseudopollen	0.9–1.1
O	Bilocular	1.0–1.3

## Data Availability

The original contributions presented in this study are included in the article. Further inquiries can be directed to the corresponding authors.
